# Supplementary material for: The Small RNA Universe of Capitella teleta
Source: Front Mol Biosci. 2022 Feb 25;9:802814. doi: 10.3389/fmolb.2022.802814 (PMC8915122; doi:10.3389/fmolb.2022.802814)
Supplement: Supplementary file 1 [file DataSheet1.ZIP › Supplement/known/CAPTEscaffold_296_17128.pdf]

Provisional ID	:	CAPTEscaffold_296_17128
Score total	:	19245.9
Score for star read(s)	:	3.9
Score for read counts	:	19236.3
Score for mfe	:	1.1
Score for randfold	:	1.6
Score for cons. seed	:	3
Total read count	:	37743
Mature read count	:	37701
Loop read count	:	13
Star read count	:	29

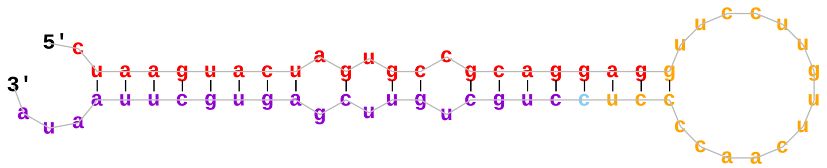

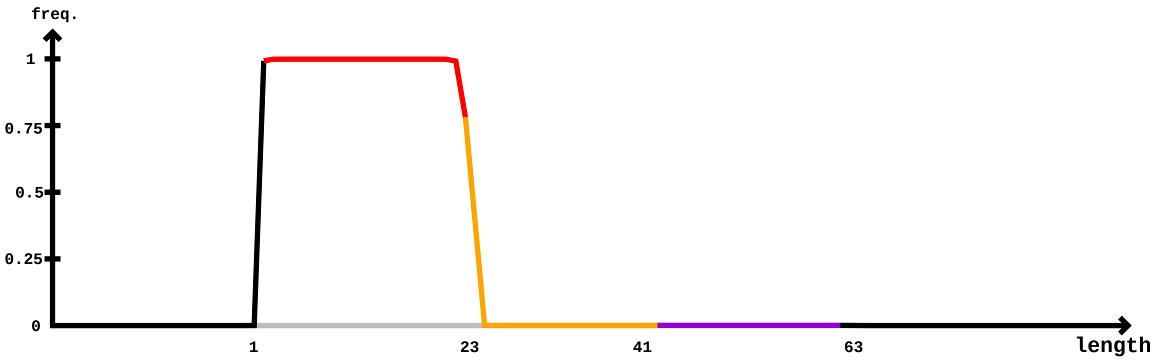

Mature **Star**

```
5'- augcucgcucauugccaaugcuaaguacuagugccgcaggagguuccuuguucaaccccuccugcuguucgagugcuuaauaucacauuugagcauuuccucucuauua  -3'   obs
    augcucgcucauugccaaugcuaaguacuagugccgcaggagguuccuuguucaaccccuccugcuguucgagugcuuaauaucacauuugagcauuuccucucuauua        exp
    augcucgcucauugccaaugcuaaguacuagugccgcaggagguuccuuguucaaccccuccugcuguucgagugcuuaauaucacauuugagcauuuccucucuauua        known
    ......(((((......(((.((((((((.(.((.((((((((.............)))))))).)).).)))))))).)))......)))))................    reads    mm    sample

    ..................Ucuaaguacuagugccgcagg......................................................................    1        1     seq
    ..................gcuaaguacuagugAcgcagga.....................................................................    1        1     seq
    ..................gcuaaguacuagugccgcagga.....................................................................    3        0     seq
    ..................Acuaaguacuagugccgcagga.....................................................................    1        1     seq
    ..................Ucuaaguacuagugccgcaggag....................................................................    1        1     seq
    ...................cuaaguacuagugccgcag.......................................................................    12       0     seq
    ...................cuaaguaAuagugccgcagg......................................................................    1        1     seq
    ...................cuaaguacuagugccgcagA......................................................................    1        1     seq
    ...................cuaaguacuagugUcgcagg......................................................................    1        1     seq
    ...................Auaaguacuagugccgcagg......................................................................    1        1     seq
    ...................cuaaguacuagugccgcagg......................................................................    250      0     seq
    ...................cuaaguacuagugccgcUgg......................................................................    1        1     seq
    ...................cuaaguacuagugcUgcagga.....................................................................    8        1     seq
    ...................cuaaguacuagugccgcagga.....................................................................    7706     0     seq
    ...................cuaaguacuagAgccgcagga.....................................................................    1        1     seq
    ...................cuaaguacuagugccgAagga.....................................................................    1        1     seq
    ...................cuaaguacuagugcAgcagga.....................................................................    3        1     seq
    ...................Uuaaguacuagugccgcagga.....................................................................    4        1     seq
    ...................cuaaguacuagugccgcaCga.....................................................................    2        1     seq
    ...................cuaaguacuagugccgUagga.....................................................................    1        1     seq
    ...................cuaaguacuagugccgcaggG.....................................................................    9        1     seq
    ...................cuaaguacuagGgccgcagga.....................................................................    1        1     seq
    ...................cuaaguacuagugccgcaggU.....................................................................    5        1     seq
    ...................cuaaguacuagCgccgcagga.....................................................................    7        1     seq
    ...................cuaaguacuagugccCcagga.....................................................................    1        1     seq
    ...................cuaaguacuagugccgcaggC.....................................................................    6        1     seq
    ...................cuaaguacuagugccgcagAa.....................................................................    1        1     seq
    ...................cuaaguacuagugGcgcagga.....................................................................    1        1     seq
    ...................cuaaguaAuagugccgcagga.....................................................................    1        1     seq
    ...................cuaaguacuaguCccgcagga.....................................................................    2        1     seq
    ...................Guaaguacuagugccgcagga.....................................................................    1        1     seq
```

```
                    Mature                Star
augcucgcucauugccaaugcuaaguacuagugccgcaggagguuccuuguucaaccccuccugcuguucgagugcuuaauaucacauuugagcauuuccuucucuauua
....................cuaaguaGuagugccgcagga.....................................................................	112	1	seq
....................cuGaguacuagugccgcagga.....................................................................	1	1	seq
....................cuaaguGcuagugccgcagga.....................................................................	2	1	seq
....................cuaaguacuagugAcgcagga.....................................................................	4	1	seq
....................cuaagCacuagugccgcagga.....................................................................	1	1	seq
....................cuaagAacuagugccgcagga.....................................................................	5	1	seq
....................cuaaAuacuagugccgcagga.....................................................................	1	1	seq
....................cuaaguacuagugccUcagga.....................................................................	1	1	seq
....................cuaaguacuagugccgcaAga.....................................................................	8	1	seq
....................cuaaguacuagugccgcGgga.....................................................................	3	1	seq
....................Nuaaguacuagugccgcagga.....................................................................	2	1	seq
....................cAaaguacuagugccgcagga.....................................................................	5	1	seq
....................cuaGguacuagugccgcagga.....................................................................	2	1	seq
....................Auaaguacuagugccgcagga.....................................................................	5	1	seq
....................cuaaguacuaCugccgcagga.....................................................................	1	1	seq
....................cuaaguacAagugccgcagga.....................................................................	3	1	seq
....................cuaGguacuagugccgcaggag....................................................................	3	1	seq
....................cuaaguacuagugccgAaggag....................................................................	5	1	seq
....................cuaaguacuagugccCcaggag....................................................................	1	1	seq
....................cuaaguacuGgugccgcaggag....................................................................	6	1	seq
....................cuaaguUcuagugccgcaggag....................................................................	4	1	seq
....................cuaaguacuagugccAcaggag....................................................................	2	1	seq
....................cuaaguacuagugcUgcaggag....................................................................	10	1	seq
....................cuaagNacuagugccgcaggag....................................................................	1	1	seq
....................cuaaguacuagugccgcaggCg....................................................................	1	1	seq
....................cuaagAacuagugccgcaggag....................................................................	14	1	seq
....................cuaagCacuagugccgcaggag....................................................................	5	1	seq
....................cuaaguacuaCugccgcaggag....................................................................	4	1	seq
....................Nuaaguacuagugccgcaggag....................................................................	10	1	seq
....................cuaaguacuUgugccgcaggag....................................................................	1	1	seq
....................cuaaguacuagugccgcaggaC....................................................................	2041	1	seq
....................cuaaguacuagugUcgcaggag....................................................................	5	1	seq
....................cuaaguacuagugccgcaggaA....................................................................	19	1	seq
....................cuaaguacuagugccUcaggag....................................................................	1	1	seq
....................cuaaguacuagAgccgcaggag....................................................................	14	1	seq
....................cuaaguacuagugccgcaggaU....................................................................	810	1	seq
....................cuaUguacuagugccgcaggag....................................................................	1	1	seq
....................cuaaguacuagugccgcaggag....................................................................	24774	0	seq
....................cuaaguacCagugccgcaggag....................................................................	3	1	seq
....................cuaaguacuagugccgcaggUg....................................................................	3	1	seq
....................cuaaguacuagugcAgcaggag....................................................................	8	1	seq
....................cuaaguacuaAugccgcaggag....................................................................	3	1	seq
....................cuaaguacuagugccgUaggag....................................................................	9	1	seq
....................cuaaguacuagugccgcaAgag....................................................................	29	1	seq
....................cuaaguacuagugccgcagUag....................................................................	1	1	seq
....................cuGaguacuagugccgcaggag....................................................................	6	1	seq
....................cuaaguacuagugccgcUggag....................................................................	1	1	seq
....................cuaaguaUuagugccgcaggag....................................................................	5	1	seq
....................cuaaguacuaguUccgcaggag....................................................................	1	1	seq
....................cuaaguacuagCgccgcaggag....................................................................	3	1	seq
....................cuaaguGcuagugccgcaggag....................................................................	2	1	seq
....................cuaaguacuagugccgcaUgag....................................................................	1	1	seq
....................cuaaguacuaguCccgcaggag....................................................................	3	1	seq
....................Auaaguacuagugccgcaggag....................................................................	22	1	seq
....................cuaaguacuagugccgcaggGg....................................................................	7	1	seq
....................cuaaguaGuagugccgcaggag....................................................................	1	1	seq
....................cuaaguacuagGgccgcaggag....................................................................	2	1	seq
....................cuaaAuacuagugccgcaggag....................................................................	1	1	seq
....................cuaaguacGagugccgcaggag....................................................................	2	1	seq
....................cCaaguacuagugccgcaggag....................................................................	1	1	seq
....................cuaaguacuagugAcgcaggag....................................................................	12	1	seq
....................cuaaguacuagugccgcCggag....................................................................	1	1	seq
....................cuaaguacAagugccgcaggag....................................................................	7	1	seq
....................cuaCguacuagugccgcaggag....................................................................	1	1	seq
....................cuaaguacuagugccgcaggNg....................................................................	1	1	seq
....................cGaaguacuagugccgcaggag....................................................................	3	1	seq
....................cuaaguacuaguAccgcaggag....................................................................	5	1	seq
....................cuaaguacuagugccgcagAag....................................................................	4	1	seq
....................cuaaguaAuagugccgcaggag....................................................................	3	1	seq
....................cuaaguacuagugccgcGggag....................................................................	6	1	seq
```

Mature **Star**

```
augcucgcucauugccaaugcuaaguacuagugccgcaggagguuccuuguucaaccccuccugcuguucgagugcuuaauaucacauuugagcauuuccuucucuauua
....................cAaaguacuagugccgcaggag....................................................................  30    1  seq
....................cuaaguacuagugcGgcaggag....................................................................  1     1  seq
....................Guaaguacuagugccgcaggag....................................................................  2     1  seq
....................Uuaaguacuagugccgcaggag....................................................................  15    1  seq
....................cuaaguacuagugccgcaggaCg...................................................................  2     1  seq
....................cuaaguacuagugccgcaggagU...................................................................  1046  1  seq
....................cuaaguacuagugccgcaggagC...................................................................  1     1  seq
....................cuaaguacuagugccgcaggagA...................................................................  245   1  seq
....................cuaaguacuagugccgcaggagg...................................................................  28    0  seq
....................cuaaguacuagugccgcaggagUu..................................................................  21    1  seq
....................cuaaguacuagugccgcaggagUuu.................................................................  2     1  seq
.....................uaaguacuagugccgcagg......................................................................  2     0  seq
.....................uaaguaAuagugccgcagga.....................................................................  1     1  seq
.....................uaaguacuagugccgcagga.....................................................................  14    0  seq
.....................uaaguacuagugccgcaggaC....................................................................  3     1  seq
.....................uaaguacuagugccgcaggaU....................................................................  3     1  seq
.....................Caaguacuagugccgcaggag....................................................................  1     1  seq
.....................Aaaguacuagugccgcaggag....................................................................  2     1  seq
.....................uaaguacuagugccgcaggag....................................................................  132   0  seq
.....................uaaguacuagugccgcGggag....................................................................  1     1  seq
.....................uaaguacuagugccgcaggagU...................................................................  6     1  seq
.....................uaaguacuagugccgcaggagA...................................................................  2     1  seq
.....................Aaaguacuagugccgcaggagg...................................................................  1     1  seq
.....................uaaguacuagugccgcaAgagg...................................................................  1     1  seq
.....................uaaguacuagugccgcaggagg...................................................................  52    0  seq
.....................uaaguacuagugccgcaggagguuU................................................................  1     1  seq
......................aaguacuagugccgcagga.....................................................................  1     0  seq
......................aaguacuagugccgcaggag....................................................................  4     0  seq
......................aaguacuagugccgcaggaggu..................................................................  1     0  seq
..........................................guuccuuguucaaccccu..................................................  13    0  seq
...........................................................uccugcuguucgagugcuuaau.............................  1     0  seq
............................................................ccugcuguucgagugcuuaa..............................  1     0  seq
............................................................ccugcugAucgagugcuuaaua............................  1     1  seq
............................................................ccugcuguucgagugcuuaaua............................  3     0  seq
.............................................................cugcuguucgagugcuuaa..............................  3     0  seq
.............................................................cugcuguucgagugcuuaau.............................  7     0  seq
.............................................................cugcuguucgagugcuuaaua............................  13    0  seq
```
